# Investigation and mapping of geological construction materials in parts of chemoga river sub basin, debre markos, Ethiopia

**DOI:** 10.1016/j.heliyon.2023.e13784

**Published:** 2023-03-07

**Authors:** Mulusew Minuyelet Zewdie, Dawit Asmare

**Affiliations:** Civil Engineering, Institute of Technology, Debre Markos University, P.O.Box 269, Debre Markos, Ethiopia

**Keywords:** Geomaterials, Geomaterials potential, Geomaterials quality, Geomaterials accessibility, Workability, Chemoga river

## Abstract

Currently the demands of geomaterials are intensively increasing all over the country regarding to the expansions of towns for the consumption of engineering structures and buildings. Therefore, searching workable, serviceable and accessible geomaterials nearby areas is a vital subject to stabilize the inflation of geomaterials. The present research was carried out in the Chemoga river sub basin in the Southern direction of Debre Markos town, North Western Ethiopia. To meet out the objectives, detailed surface explorations were carried out through systematically selected travel lines (across the strike and along streams) to detect the different lithologic formations and the contacts; A number of representative rock samples were collected for visual interpretations; GPS reading and metallic meter were the materials used in the field to quantify stratigraphic units, bedding thickness and lamination the lithologic formations; The Brunton compass was used in the field to measure the orientation (dip direction, dip amount and strikes) of geological structures. Besides to this, related literatures were reviewed to have a general background on the subject matter.

Basalt and Sandstone are the dominant lithogical units in the study area and have existed with about an average thickness of 500 *m* and 145 *m* respectively. Fresh to slightly weathered basalt and columnar basalt that used as a masonry stones are ease to access, workable and the most dominant lithologic units in the study area. Fine to coarse grain reddish to light color sandstone interbedded with siltstone and mudstone is well exposed. Based on the detail field exploration and judgments, Sandstone unit has been well exposed in a desirable potential and quality. And based on the detail field investigation and researcher judgment, the sandstone resource could answer the demands of construction materials for the surrounding areas of Debre Markos town for several thousand of years. Geometrically, mostly massive and steep rock faces are well developed which are difficult for quarrying process and required high explosive materials. Generally, a sandstone rock unit is surrounded by three major normal faults and inaccessible as compared to basalt unit. Regarding to environmental influences/effects, the area is sparsely farmed and it is not common that an extractive activity does not show any environmental and social impacts. Chert is exposed on geographically limited area and rarely observed following stream cuts. Chert and basalt units are slightly to highly weathered and showed alterations on the surface. Finally, the potential and the spatial distribution of geomaterials are investigated and revealed on the map using ArcGIS software and on the basis of the findings of the present research, recommendations are forwarded.

## 1 Introduction

Naturally gifted resources play a vital role for the fast development of a given country. Industrial minerals and rocks, economically valuable minerals (ore bodies), natural gasses and others surface and subsurface hidden treasures are some of none renewable and invaluable resources. All those assets have not fairly preserved all over the world. Their occurrence and potential is dictated by the geological processes involved on the Earth. Therefore, identifying their occurrence and investigating of potentially available and qualified geological construction materials are significantly important to make decision for quarry site selection. Site characterization is a major component of site investigation [1,2].

Geological construction materials have a wide range of application in construction, manufacturing, chemicals industries, metallic minerals (ores), non-metallic minerals, agriculture, glass, ceramics industries. Geomaterials for construction were among the first mineral raw materials exploited, processed and used by man [3]. Natural stone is one of the oldest and more durable construction materials. However, its importance for the construction industry has changed over time and so has its perception by society [4]. Basic raw geological construction materials are: crushed stones, dimension stones, gravel, aggregates light weight aggregates (perlite, pumice, scoria, vermiculite cement) clays, limestone, gypsum, sandstone etc. These are the fundamental geomaterials and required in high bulk quantity. The geologic materials discussed in this paper fall under the general category of industrial rocks and minerals. Particularly focus on rocks and minerals used for engineering construction in large volume.

Sandstone is a sedimentary origin of industrial rocks composed mainly from silicate minerals and plays a vital role for the development of national economy. Basically, the rock unit is the most consumed (required) Earth materials at any point and linear engineering structures. The crushed sandstone is used as building stones in the form of mortar and concrete. They are more commonly used in the engineering construction of buildings, dams, bridges, roads, etc. Beside to limestone and gypsum, sandstone is one of the raw geological materials used in cement industry. High potential, fresh and workable geological construction materials of sandstone and basaltic formations are most available and easily accessible in the study area.

### 1.1 Description of the study area

**Fig. 1:**
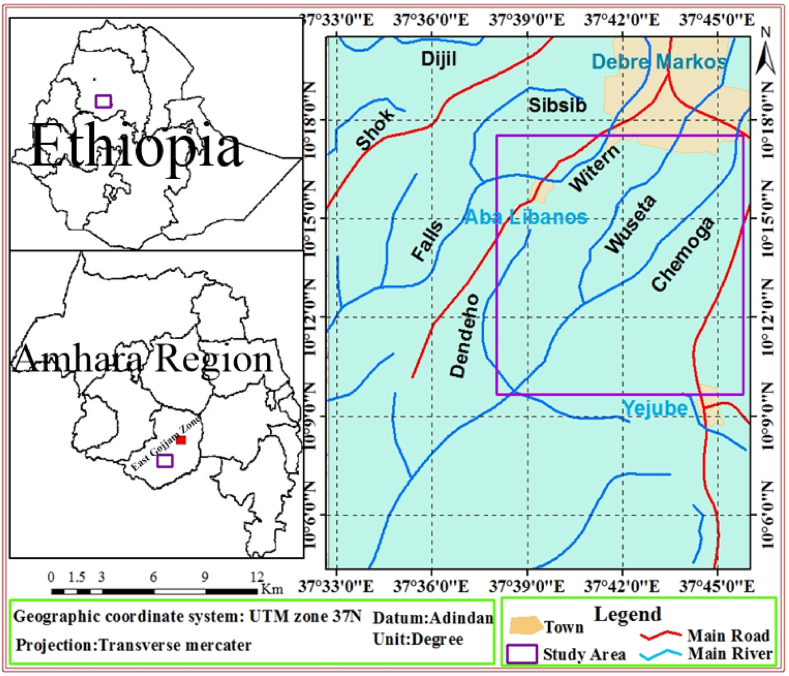
Location map.

The study area is located in North Western part of Ethiopia about 300 km far from Addis Ababa, the capital city of Ethiopia and about 17 km far from Debre Markos town in Southern direction. More specifically, it is bounded by a geographic coordination system of Universal Transverse Mercator (UTM) reading latitude 1,125,000–1,137,000 *m* north and longitude 351,000–369000 *m* east which covers an area of 216 km^2^ (Fig. 1).

The perennial rivers in the study area are called Chemoga and Wuseta which originating from the highland region of Choke Mountain and have over 45 km lifelong and flows from North to South. After incorporation of all their tributaries, finally they feed the Abay River. They revealed many times significance volume changes in a year. In summer becomes more powerful and poses environmental problems along their flow channel.

**Fig. 2:**
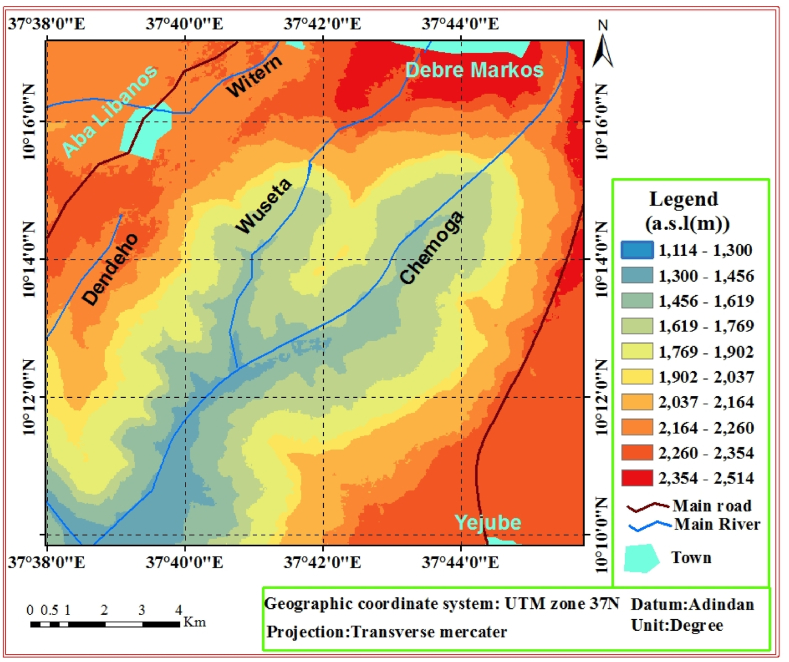
Physiographic map of the study area.

The study area has defined by amazing landforms of deeply incised valleys, cliff, rugged and some parts of the area are flat topography. These multispectral bands of surface features (land forms) are the implication of geodynamic and geomorphic processes have been acting on it. The geomorphology of the area is highly variable and it is generally the implication of repeated volcanic and geological events (i.e. erosion, flooding, deposition, landslide and others processes). The area approximately lies an average elevation of 1814 *m* and it reaches up to 2514 *m* above mean sea level (Fig. 2).

Physiography is closely associated with the geological and the geomorphological history of the environment and also effectively influences the geomechanical setting [5]. The physiography of the study area is characterized by forming a steep slope associated with benches on basalt units; while gentler slopes and topographic breaks in highly weathered rocks and residual soil deposits. Moreover, most areas are characterized by cliff landforms. The slope of the study area is defined by rugged land features which is steep at the top of the slope and becomes gentler towards the toe.

**Fig. 3:**
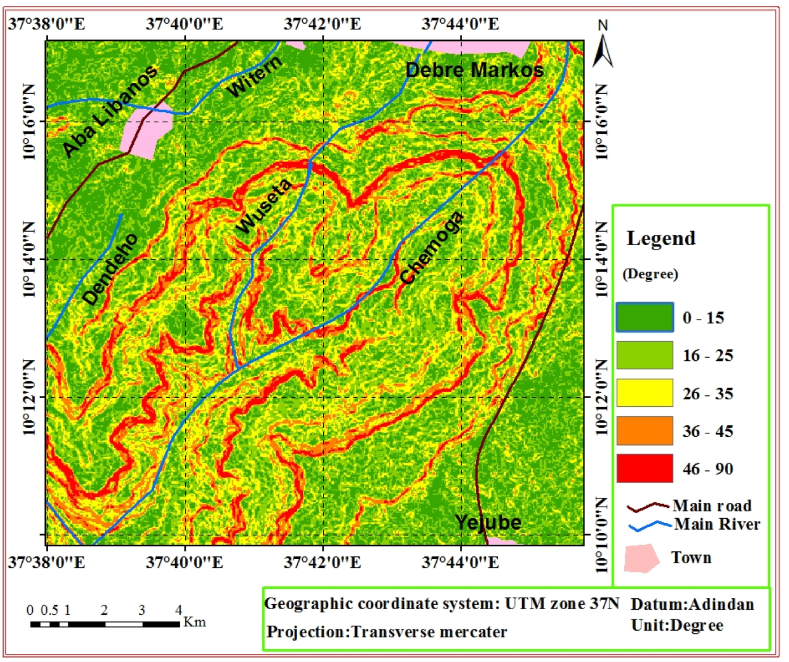
Slope map of the study area.

The slope map was initially produced from DEM 30 *m*. This was very helpful to characterize the slope geometry during practical field work. Because, it was challenging to characterize the angle and height in the field accurately due to rugged land features. Based on Anbalagan [6] slope classification, the present study area slope map was produced by classifying the slope angle into five classes (Fig. 3). Accordingly, the classes are; very gentle slope (<15°), gentle slope (16–25°), moderately steep slope (26–35°), steep slope (36–45°) and escarpment/cliff (>45°) (Fig. 3).

**Fig. 4:**
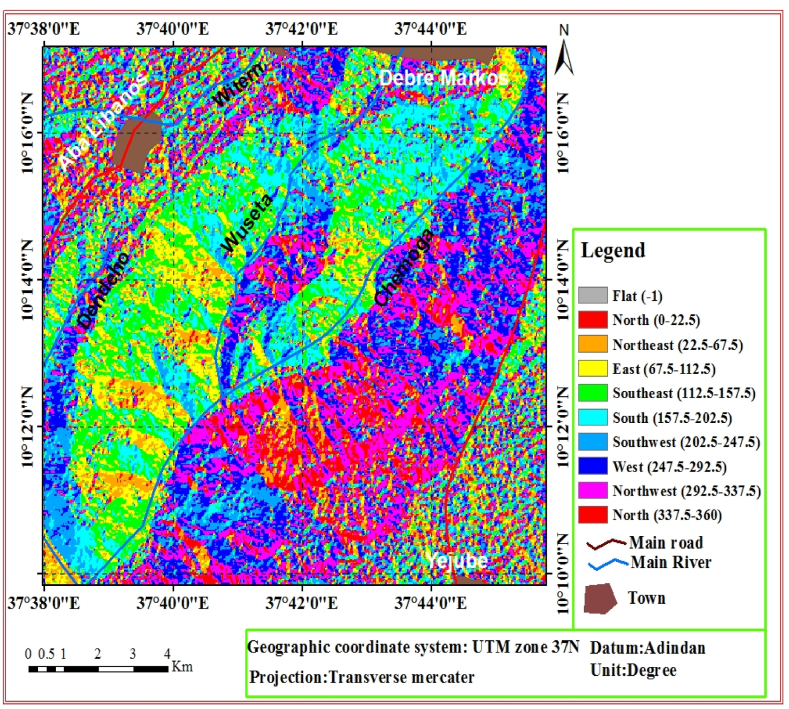
Aspect map of the study area.

Slope aspect refers to the direction in which the slope is facing. The aspect of slopes in the present study area was derived from the DEM 30 *m* and it was classified accordingly Hamza and Raghuvanshi [7] classification system into 10 classes. Flat (−1°), North (0–22.5°), Northeast (22.5–67.5°), East (67.5–112.5°), Southeast (112.5–157.5°), South (157.5–202.5°), Southwest (202.5–247.5°), West (247.5–292.5°), Northwest (292.5–337.5°) and North (337.5–360) (Fig. 4).

**Fig. 5:**
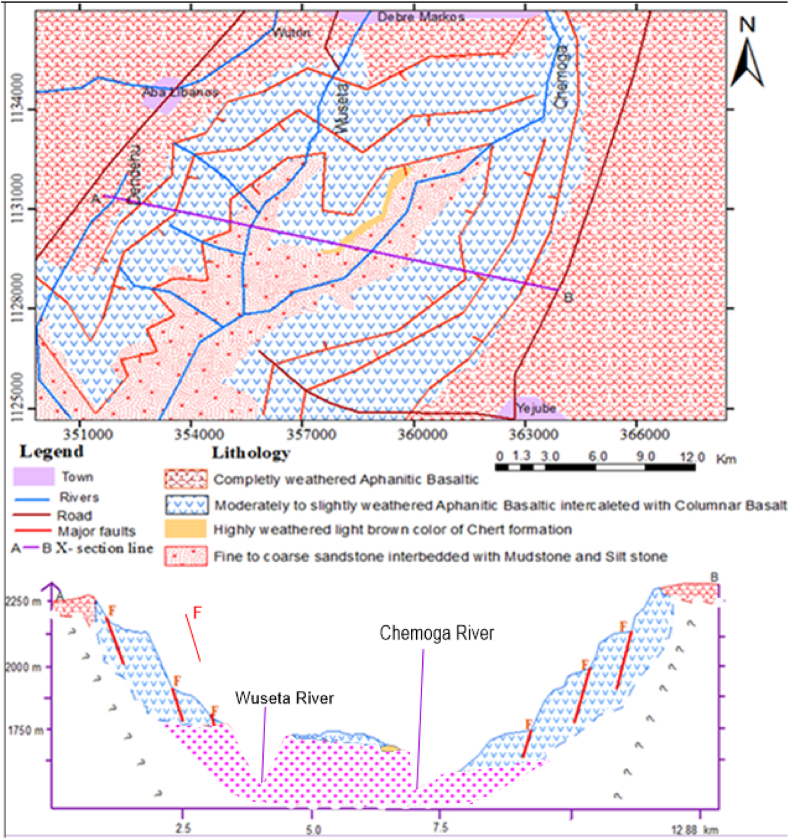
The geological and x-section map of the study area produced using ArcGIS software.

**Fig. 6:**
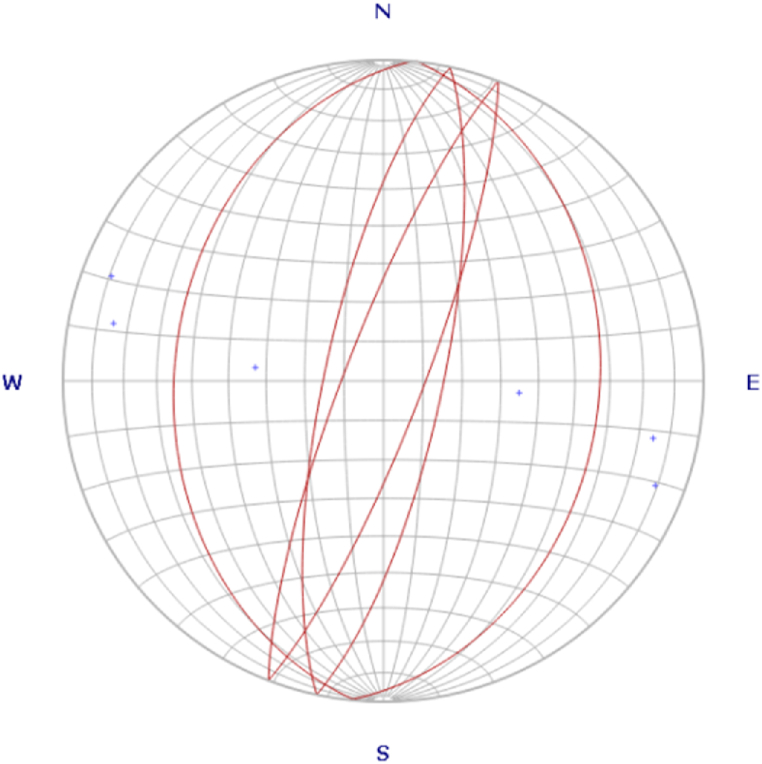
The six major normal faults plotted on Rose diagram and they illustrated a mirror reflection to each other.

**Fig. 7:**
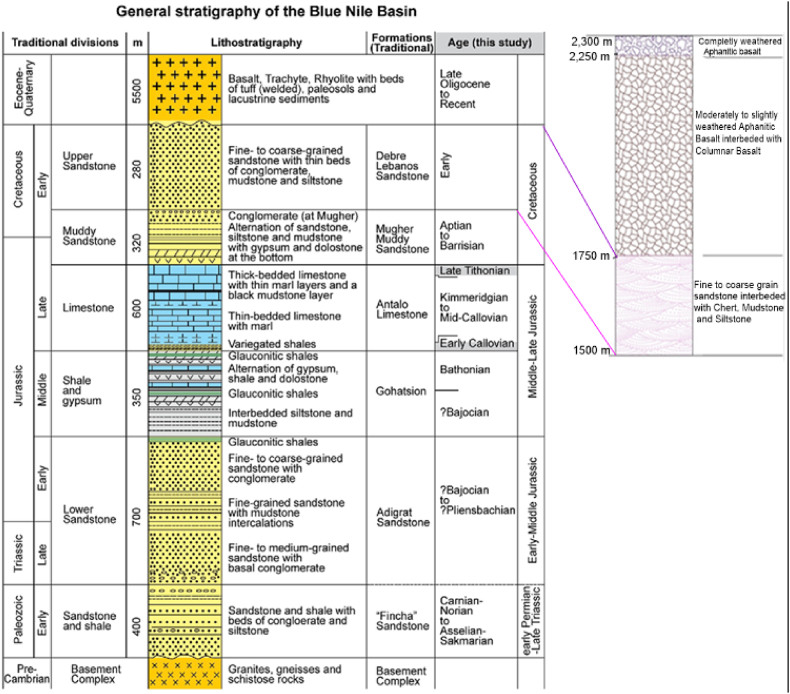
The correlation of local geology (Fig b) to the generalized stratigraphic units of the Blue Nile Basin (Fig a) [21].

**Fig. 8:**
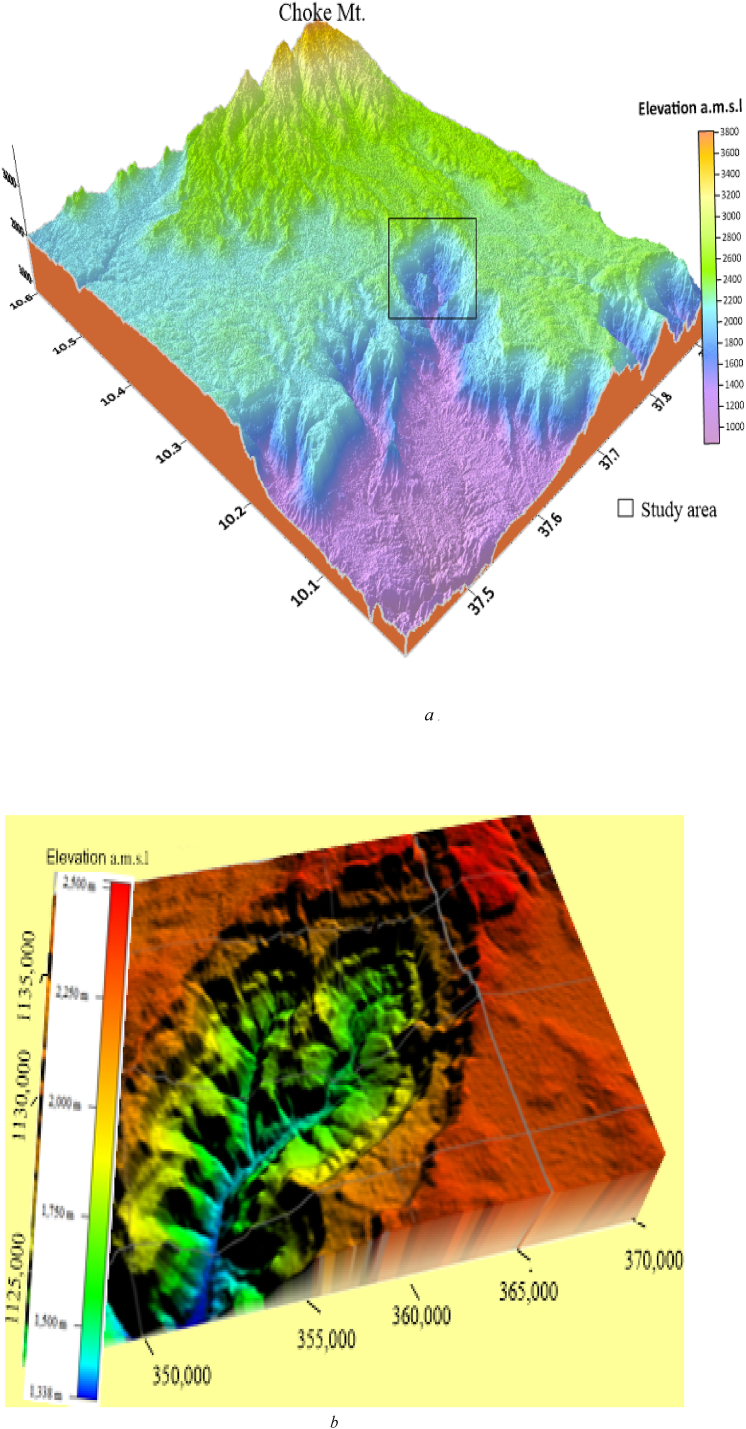
(a) Regional 3D view of the study area using surfer softwares.(b). Local 3D view of the study area using global mapper softwares with 1.5 times the vertical exaggeration.

### 1.2 Objectives and rationales of the study

The demand of geological construction materials is intensively increased all over the nation for the consumptions of abruptly expanded engineering projects. Almost all contractors and communities around Debre Markos town are transported geological construction materials from remote area and Abay river basin which is about 110 km far. In some situation they are enforced to use low quality geomaterials. Therefore, investigating of potential and qualified geological construction material nearby areas is extremely important issue to resolve those problems. The main objective of the study was to investigate and map the geological construction materials following Chemoga river basin by surface exploration through selected travel lines.

**Chart 1:**
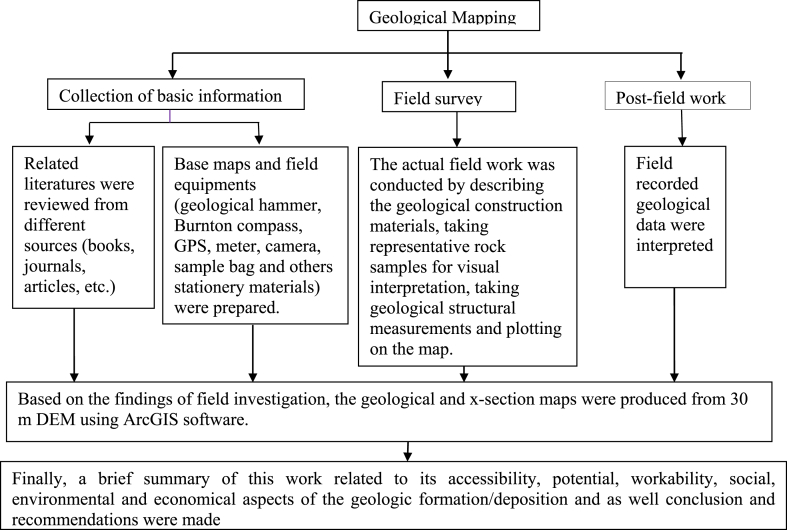
General method that were followed for the present study.

Surface exploration was carried out following systematically selected traverse lines (across the strike and along streams) to detect the different outcrops and geologic structures. Geological hammer, Brunton compass, meter, Geographic Position System (GPS) and digital camera were tools used in the field to sample or to collect fresh representative rock samples for visual interpretation; to measure the orientation of geological structures (dip and dip direction and spacing of discontinuities); to measure the thickness of lamination and bedding; used to locate the exact position on the ground and used to take a photo of different geological features that emphasize the actual condition of the site respectively. Finally, the geological and geological cross-section map, topographic, contour, aspect, slope and others maps were prepared from high precision of 30 *m* digital elevation model (DEM) using mostly ArcGIS software. Therefore, to achieve the proposed problem, the procedure or steps followed were stipulated below (Chart 1).

## 2 Geological environment and geomaterials

### 2.1 Geological environment

The economic histories, civilization and culture of the well developed countries are exclusively depends on the credits and debits of the geological environment. Thus, exploration and the use of earth resources initially started in the countries of naturally donated geological environments.

**Chart 2:**
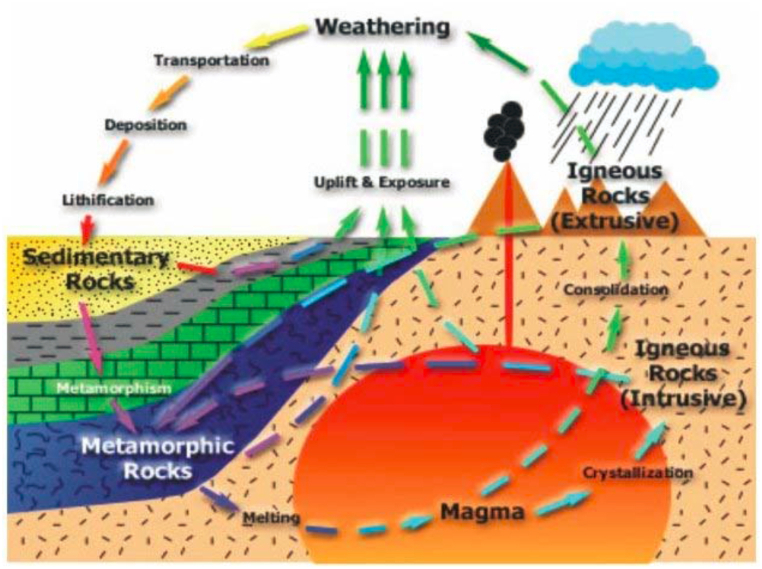
Rock Cycle (from National Science Teachers Association web site http://www.minsocam.org/MSA/K12/rkcycle/rkcycleindex.html).

The three major classes of geological environments are metamorphic, igneous and sedimentary terrains. By recognizing the geological process one can search for required geological material [8]. Mineral and rock formations and their associations are dictated by geologic environment and the processes involved in the earth. Rocks are continuous changed from one form to the other due to the different geologic processes acting on them (Chart 2). The geological processes have their own impact on the mineral composition and properties of rocks, engineering characteristics of rocks and environmental changes as a whole. Rocks and their formation processes are the fundamental topics need to understand them in order to understand Earth's history [9]. All industrial minerals and rocks, economically valuable minerals and rocks are all the direct products of geological processes. According to the description of natural processes of rock origin and occurrences, igneous rocks those are crystallized and consolidated directly from magma and is exposed for erosion, resulting in sediment which gets buried, lithified in to sedimentary rock, which is either eroded or buried deeper and metamorphosed; the metamorphic rock can be buried further and re-melted, or exposed and eroded into sediment (Chart 2). Igneous rocks which are formed directly from magma below the surface (intrusive) and on the earth's crust (extrusive) are subjected for physical disintegration and chemical decomposition of rocks. Then after can be eroded and transported by any means of transporting agents and deposited layer by layer and subsequently lithified into solid rock to form sedimentary rocks. Metamorphic rocks are formed where both igneous and sedimentary are transformed under heat and pressure. In a general speaking, igneous and metamorphic rocks are denser, harder and stronger, whereas sedimentary rocks are more porous, and sometime less durable as a result they have less application as a masonry stone.

For the consumptions of radically escalating engineering projects works, the pressure to discover new resources (i.e geological construction materials) is significantly important [10]. The extraction of geological construction materials for building construction, has taken place since time immemorial [11]. On the present day plenty of researchers and investors have introduced to the country for investigating and exploring those economically valuable ore deposits and industrial minerals and rocks.

Ethiopia is rich in surficial and subsurface treasures. Basically, in Ethiopia, there are five major basins filled with sedimentary origins of industrial minerals and rocks. Those are Ogaden, Abay, Tigray, Ommo and Gambela basin and they are the main sources of raw materials for cement and gypsum industry. Currently the governmental policy provides extreme attention in the area of exploring and extracting the resources to enhance the people's daily lives and striven to build core technologies those can mobilize the competitiveness of the nation and everlasting productivity.

### 2.2 Geomaterials

Geomaterials are extracted from the upper parts of the Earth's crust and are used for construction purposes in a wide range of applications. Geomaterials suitable for buildings and infrastructures represent a broad group of mineral raw materials that were formed by various genetic processes, reveal diverse properties and thus are suitable for many applications [3]. Many naturally occurring Earth materials, such as clay, sand, aggregates, crushed stone and dimension stones are used in the construction of commercial and residential buildings.

**Aggregates:** they are produced from two main sources, crushed rock and sands and gravels deposits. Crushed rock aggregates are produced from hard, strong rock formations [12]. Aggregate can be classified as concrete aggregate, road aggregates, riprap and other large rock material.

**Dimension stone:** It is any rock material that is cut and shaped into specific sizes to be used for retaining walls, blocks, slabs and for other purposes. The locally availability and requires little energy for extraction and processing of the dimensional stones make, to be used since the beginning of civilization. Depending on the geological environment and characteristics of geological formations, dimension stones are separated from their natural bedrock formations using different techniques (excavation, blasting, heating, wedging) and processed for their use in engineering works. **Excavation:** This technique is engaged when stones to be quarried are lying buried in earth or are under loose overburden before excavating.

**Blasting:** It is the quarrying of stones using different explosives materials depending upon the characteristics of the rocks to be quarried.

**Wedging**: This method is suitable for quarrying rocks subjected for a set of discontinuities. The operation is started near a vertical face.

**Heating:** This method is favorable where only small blocks of more or less regular shape are required and suitable rocks bedded in horizontal layers, which have not much thickness to be quarried.

In the case of sedimentary rocks, where beds dip steeply, rocks can pose slope instability problems when excavated. On the other hand, if beds of rock dip gently, it is helpful to develop the quarry floor along the bedding planes. The massive nature of igneous and metamorphic rocks, quarry can be developed in any direction depending upon the environmental condition and the specification of the planning.

#### 2.2.1 Factors determining the choice of proper geomaterials for engineering work

The design of building structures and elements must be adapted to the mineralogical, physical and mechanical properties of stone [4]. The physical, geotechnical, mechanical and chemical properties of geomaterials are among many governing factors for the selection of geomaterials for construction. Geomaterials composed from chemically active elements can readily lose their strength and quality. For example Carbonate rocks composed of mainly from CaCO_3_ (Limestone, dolomite) can react with acidic rain and remove the dissolved fine clay particles. Subsequently weaken the strength of the rock and this process is common both on and below the surface. On the other hand, most of sedimentary and other rocks lack of networked grain to grain bonding and can be easily broken or fail its strength. Coarse grain sandstone exhibit rounded grains and have no networked grain to grain bonds. Therefore, the geomaterials to be selected for engineering structures have to fulfill structural requirements, less weathering susceptibility and reveal more durable, impermeable, non porous, high crushing strength, hard, stiff and strong for the internal and external impacts, workable and aesthetically attractive. The course of weathering of exposed rock surfaces depends on both internal factors, such as mineral composition, structure and texture of the rock considered, and external ones, for instance, climate and anthropogenic pressure [13]. Thus, mineral compositions, texture, structures, porosity, permeability, durability, strength of rock, resistance to fire are the governing factors of geomaterials for engineering structures**.**

Generally rocks such as basalt suitable for construction works except as building stone due to their dark color they are not pleasant to be used as building stone in face work. Quartzite suitable for construction works except as building stones because of its non-workability. Well cemented angular grains and quartz composition (chemically inert) characteristics of the Siliceous sandstones made them to be used in any engineering works such as building stone, as site of foundation, road stone, railway ballast, and for tunneling while the argillaceous sandstone is not applied in any engineering works due to clayey matter on contact with water forms weak and lubricating matter that causes a slippery base. Shale is unsuitable as construction material due to their incompetent and hydrophilling nature. Mostly limestone is not applicable as foundation rock due to its solubility nature. Conglomerate is not applied in any engineering works due to their incomplete cementation, heterogeneity of grains, smooth face and roundness of the grains.

Because of their massive, dense, competent, inter locked texture, non porous, impermeability, high crushing strength, good polishing, resistant to fire, frost and abrasion, workability, aesthetically attractive nature of some igneous rocks (Granite, ignimbrites, Rhyolite, dacite, andesite), metamorphic rocks of schist and gneisses (i.e granite schist and gneisses, quartzite, marble) and sedimentary rocks (compacted siliceous sandstone, chert, dolomite) are applicable in engineering buildings.

### 2.3 The fundamental features of earth materials for construction

**Chart 3:**
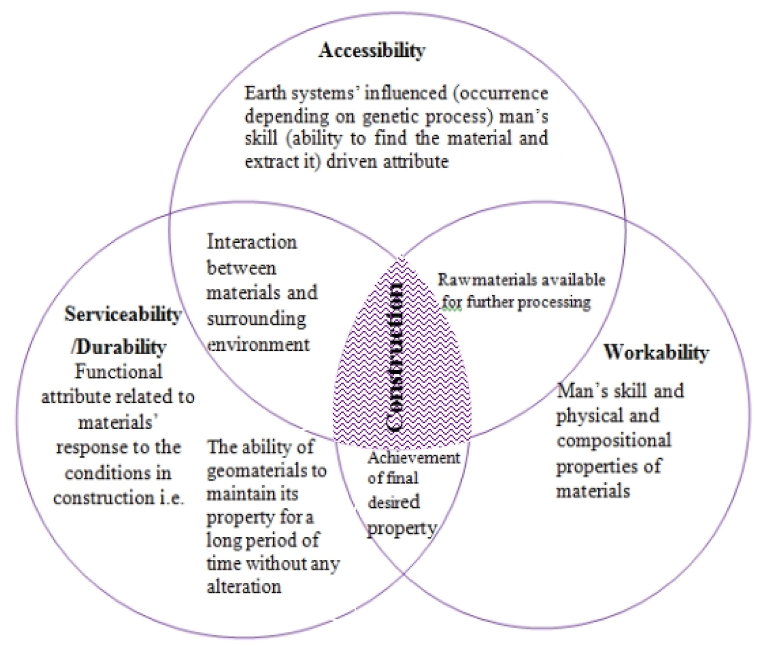
The relationship of serviceability, workability and accessibility of geomaterials in construction with little modification from [3].

Geomaterials to be used in construction, it should fulfill the three entities/conditions at the same time; easily available, workable and Serviceable (Chart 3 [3]).

**Accessibility:** The sources of geomaterials that have been formed by various genetic processes in the Earth's crust can be obtainable. However, in some parts of the study area the geomaterials (columnar basalt, basalt and sandstone formation) are not easily ease of use (reachable) due to the natural barrier of cliff and deeply incised gorges.

**Workable:** It the skill of humans to transform the raw materials into new specific size and shapes by applying certain technologies. Workability is primarily driven by man's skills to process material once extracted from its bed rock formation to attain desired composition, physical properties and dimensions. The feasibility of geomaterials is directly governed by the physical and chemical properties of the rock.

**Durable/serviceable:** It is the ability of geomaterials to uphold its property without significant change for a long period of time. This quality is absolutely restricted by mutual interactions among mineral composition, its properties, mode of processing and environmental conditions. Generally the relationship of serviceability, workability and accessibility of geomaterials in construction is illustrated below on (Chart 3). Based on geological field investigation, potentially and qualitatively desired geomaterials for construction purpose are investigated. Workable, serviceable and accessible sandstone and volcanic rocks of basaltic formations are exposed with required potential and quality for engineering construction purposes.

### 2.4 Geomaterials excavations/quarrying

The process of breaking and obtaining building stones from their natural rock outcrops is excavation or quarrying. If the required material is available in sufficient quality and quantity from the surface mostly open quarrying can be applied. Depending on the environmental condition and geological formation, quarrying is done by one of the following four techniques (i.e. by excavating, blasting, heating, wedging) after investigation of its quality, quantity and economic benefit. Dimension stone, crushed stone and broken stone are the main products in quarrying process.

Accidents in quarry sites mostly are due to falls of the over burden or slides of the rock slopes and mishandling of explosives. So, in quarry sites special consideration should be considered to minimize the geological hazards caused by the disturbance of quarrying processes.

### 2.5 Geological setting

The study area is found in the Northwestern plateau of Ethiopia, which is characterized by the geology of the Blue Nile basin and its evolutional development have being engaged the attention of plenty of researchers [[14], [15], [16], [17]] & others). All the above listed researchers and others (not listed here) have carried out their study on the stratigraphic and structural evolution of the Blue Nile Basin and have come upon a common idea. According to Gani et al. [17] the Blue Nile Basin has evolved in three main phases.

1) Pre-sedimentation phase: include pre-rift peneplanation of the Neoproterozoic basement rocks, during Palaeozoic time;
2) Sedimentation phase: from Triassic to Early Cretaceous;
3) The post-sedimentation phase:

Chemoga River is sub basin of Blue Nile basin developed by geological processes in the past. The basin is filled by marine deposit by the repetitive transgression and regression processes of the Indian Ocean. The basin is potential sources of geomaterials and can be quarried and mined from their bedrock occurrences. Durable, structurally strong, easily worked and accessible, attractive and qualified geomaterials are the fundamental elements of geological construction materials. They are used by the society to fortify the development of a nation.

According to Matebe et al. [18], the regional geological environment of Debre Markos area has been mapped as both Igneous and Sedimentary rocks of varying chronological time scale of Quaternary to Paleozoic era. The regional area is covered by igneous rocks of middle and lower basalts and pyroclastic rocks of tertiary formation and as well as sedimentary origin of the sandstone of Paleozoic to Mesozoic formation. More specifically, the study area is predominantly covered by sedimentary origin of upper Sandstone (Reddish brown, medium to thickly bedded, strongly cross bedded sandstone) and Sandstone (Reddish brown, slightly cross bedded).

### 2.6 Local geology

The local area is covered mainly by basalt and sandstone rock units. Moreover, geographically limited different lithologic units such as Chert, Shale, Siltstone and Mudstone have been investigated. Those are mostly well exposed along stream gorges and road cuts. The different exposures of lithologic units in the study area are explained as follows.

#### 2.6.1 Alluvium sediment

**Plate 1:**
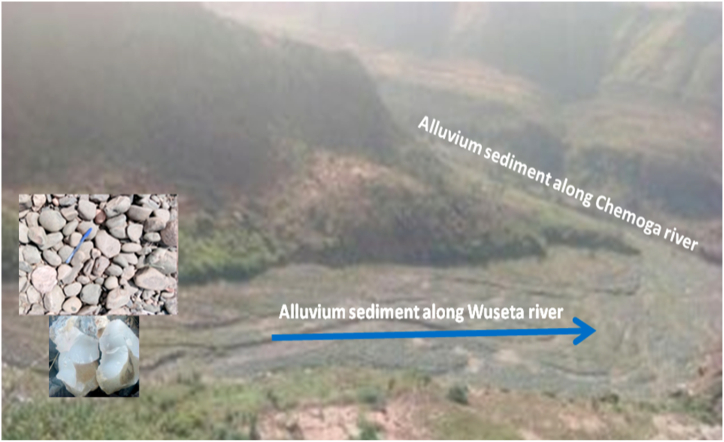
Different sizes and shapes of unconsolidated cataclastic sediments along Wuseta River.

The alluvial deposits are mostly recent and are being deposited as a result of the transport of sediments from the down wash sloppy area. Different sizes and shapes of unconsolidated cataclastic sediments of breccias, conglomerate, boulders, gravels and sandy soils have (being) deposited along Chemoga and Wuseta Rivers. Angular to well round smooth faces and aesthetically attractive pebbles and boulder of conglomerate have been deposited along the channel. Those well rounded alluvium sediments signify as they have being transported from a large distance by saltation (jumping) and rolling (bottom traction) process. Breccias are locally transported alluvium sediments. Genetically different origins of conglomerates are observed as shown below in the (Plate 1). Those geomaterials are used as aggregates for concrete and as well for decoration and well packing purpose.

#### 2.6.2 Aphanitic Basalt

Aphanitic basalt is an igneous rock unit solidified by the rapid cooling of the flow of lava on the surface of the earth. Accordingly the definition of the International Union of Geological Sciences (IUGS) classification scheme and Santos and Hartmann [19], Aphanitic basalt is compositionally mafic with generally 45–53% silica (SiO_2_) and 65% of the rock is feldspar (aluminum silicates of the alkali metals Na, Ca, k, Ba …). It is the most common volcanic rock type on Earth crust.

Depending on the cooling history of the magma, rocks with similar chemical composition and minerals present could have different textures. The Aphanitic basalt is formed by rapid cooling of magma and show fine grain mineralogical textures while for the similar composition of Gabbro (intrusive) formed by slowly cooling of molten earth materials and the individual crystal grains are visible with naked eye. The fresh and weathered color of the basalt rock is light and dark grey respectively. Due to high concentrations of weathering susceptible minerals plagioclase, basaltic rocks have been distinguished as dark.

**Plate 2:**
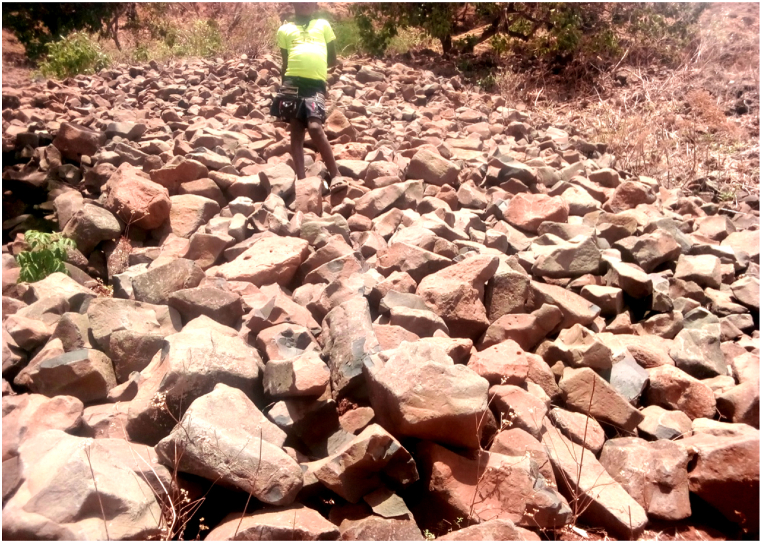
slightly weathered different sizes of colluviums rocks on the foot of cliff areas.

It is the most dominant lithologic unit. Moreover geographically limited area colluviums rocks are preserved on the foot of cliff areas. These formations are unconsolidated and randomly oriented accumulation of various sizes of rock fragments (Plate 2).

**Plate 3:**
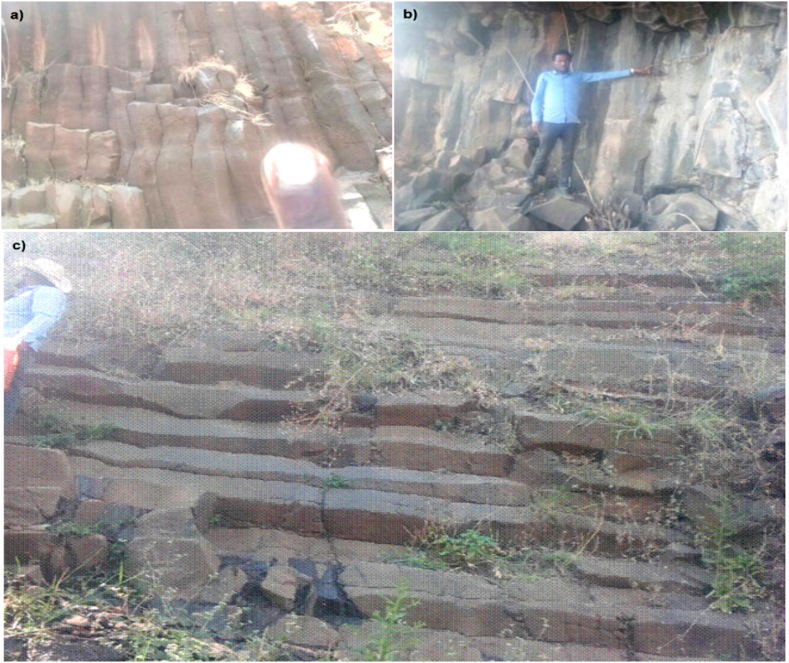
Columnar basalt exposure plate 3a & b. A vertical fresh to slightly weathered tetragonal columnar basalts well exposed on the hill sides at different location (note: on plate 3a. The index finger is not the scale). Plate 3c. Horizontally bedded fresh to slightly weathered light grey tetragonal columnar basalt formation at the hill side of Fenkatie

**Columnar Basalt:** During the cooling of a thick lava flow, the primary structures of contractional joints were formed. If a flow cools relatively rapidly, significant contraction forces build up. The geometric properties of the lateral shapes of these columns can broadly be classed as a systematic cellular network. These structures are predominantly tetragonal in cross-section as shown below on (Plate 3), but polygons with three to six sides can be observed on different location of the study area. The size of the columns depends on the rate of cooling; supper cooling magma results a small (<1 cm diameter) columns, while slowly cooling is more likely to produce large columns.

Basalt is used in a wide range of engineering construction (as crushed aggregate, building blocks, Armourstone, in the groundwork, retaining walls, riprap, and cobblestones. Generally, this rock unit has an excellent bearing capacity, durability, thermal insulator and others engineering properties.

Armourstone is a large blocks of rock that are used to protect the upstream face of dams against wave action, in the construction of river training schemes, in the river bank and bed protection and stabilization, in the prevention of scour around bridge piers, as well as for the protection of sea walls.

#### 2.6.3 Chert

Chert is fine grain and silica rich sedimentary origins of rock. Thin layers of highly weathered Chert formation are observed on the northern part of the study area at a local name of Yezegzeg. On a local scale (50 cm to several meters), road and stream cut exposure of slightly to completely weathered Chert is rarely observed in the area and have existed in a great variety of colors (from white to light grey).

It's characterized by its hardness and conchoidal fractures that create sharp edges. These properties made the Chert used in early human civilizations for creating tools and weapons. Often used as an aggregate for concrete and as a geo-material for road surfacing. It is particularly suited to road surfacing as rainwater firms.

**Plate 4:**
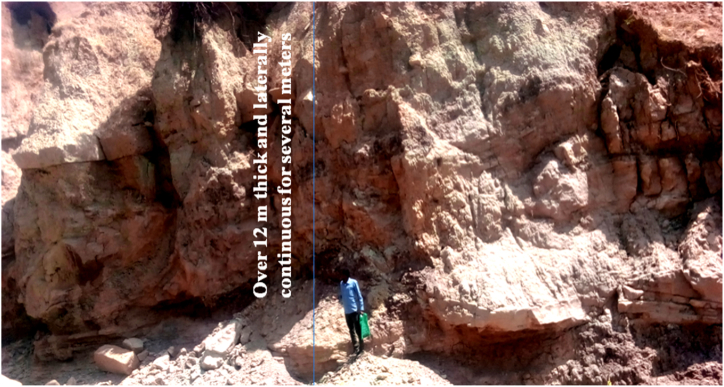
Light to reddish color of Mudstone well exposed on the local name of Argoshesh gorge at GPS reading of long. 37^ο^ 40^׳^ 40 ^״^, lat 10^ο^ 13 ^׳^ 30^״^ and alt. 1545 *m*.

Accordingly the scale used for this work, unmappable sedimentary origin of **mudstone** composed from aggregate of clay and silt-sized particles is locally deposited. The rock unit is existed in distinct quality. Aesthetically attractive, looking animal flash, soften and sticky property (Plate 4). It exists in varieties of color reddish, brown and light. The local society used it as paint to coat their residential buildings. However, the rock unit is useless in the direct applications for the construction industry; It has many applications in different industries mix up with others earth materials. It is extremely fine grain (silt and clay size) texture.

#### 2.6.4 Sandstone

Sandstone is one of the most common types of sedimentary origins of rock and is found in sedimentary basins throughout the world. It is often mined to use as a construction material or as a raw material for manufacturing industry.

Based on the stratigraphic relationship the Chemoga sandstone formation is directly correlates with Debre Libanos Sandstone (Upper Sandstone) formation of the Blue Nile basin. According to Ref. [17] sandstone was deposited in Late Jurassic to Early Cretaceous period by the 6th stages of sedimentation phase of final marine regression processes. The overall depositional environment of this unit is interpreted to be continental alluvial to fluvial. The local Sandstone is overlain by volcanic rocks and encircled by three consecutive normal faults.

Sandstone is clastic in origin and has mainly of sand sized (0.062–2 mm) mineral particles or rock fragments. The sand grains are rounded and highly spherical. Most sandstone is composed of quartz or feldspar minerals. Due to impurities (manganese, iron oxidation) within the minerals of sandstone formation, it exhibits in various colors, but more commonly observed colors are red and white.

The weathering resistance, hardness, uniformity of grain size, friability of the structure and its workability make the sandstone a common building and paving geomaterials. Predominantly the sandstone formation of Chemoga River reveals well sorted and locally comprising poorly sorted clastic textures*.*

The sandstone formation is thick to massive bedding, mostly tabular and generally found interbedded with argillaceous sandstone which contains a significant amount of silt and clay.

**Plate 5:**
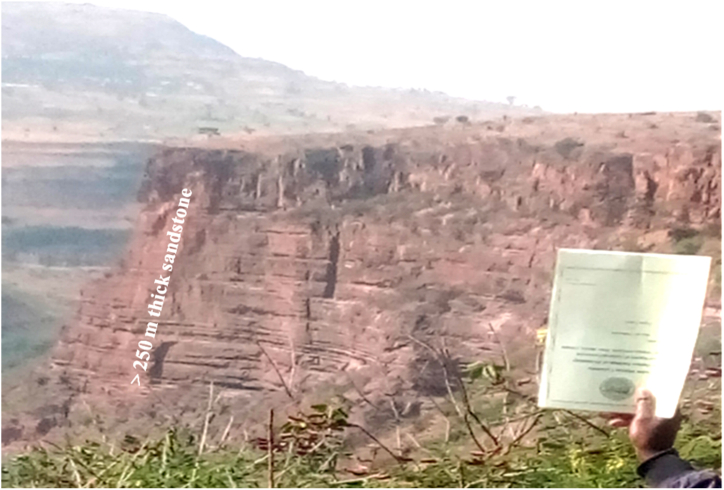
Horizontally bedded reddish Sandstone along Chemoga sub river basin at a local name of Yezegzeg and forms over 250 *m* vertical rock faces (the photo was taken at the top of the cliff and the note book doesn't represent the scale).

Locally graded bedding and unidirectional cross bedding structures are revealed on the vertical faces of sandstone exposures, but generally graded structures are not recognized. These faces are an evidence of the deposition under energetic and uniformly elevated energy of water wave action. Beds of Sandstone mainly form highly visible cliffs, deeply incised gorges and other geomorphological features (Plate 5).

Sandstone has significant applications in many industries. Primarily used with cement to make mortar for masonry work and plaster. It is also used as a part of the concrete mix and has many applications in Ceramic industry. It has been widely used around the world for temple, domestic construction and house wares since prehistoric times, and continues being used.

**Plate 6:**
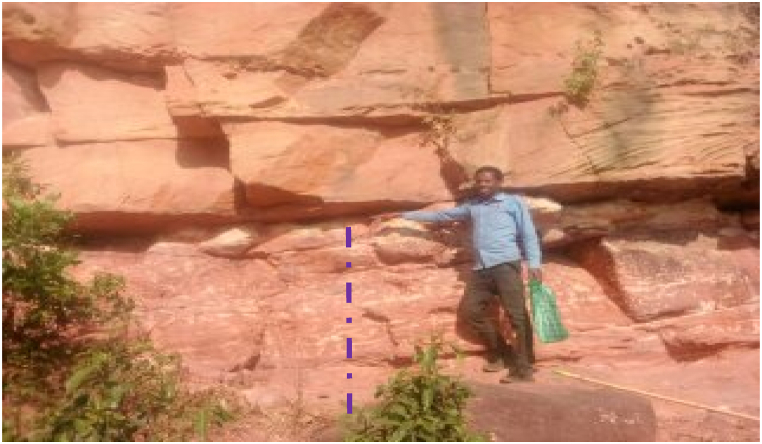
A horizontally interbedded two to 3 m thick and laterally continuous fresh to slightly weathered brown and slight rusty red Siltstone is preserved on the cliff of Wuseta River.

Additionally Unmappable, horizontally interbedded two to 3 m thick and laterally continuous fresh to slightly weathered brown and reddish Siltstone is well preserved on the cliff of the Wuseta River (Plate 6). Siltstone is sedimentary rock that formed from fine grain clastic sediments. The colors of the rocks are greatly influenced by the color of predominant mineral. The reddish and light colors of siltstone are the indication of the trace elements present in the rock, and both reddish and light colors are most often related to traces of iron and quartz respectively.

Siltstone is formed in a sedimentary or near water body environment from suspended fine grain particles where the current has continuously lost its energy. Therefore fine grain sedimentary rocks such as siltstone, mudstone and shale are deposited as progressively declination of the current energy. Thus, siltstone, mudstone and shale are interrelated rocks which are distinguished by particle size.

The geological and the cross sectional map along the cross section line of “A” to “B” was prepared using ArcGIS softwares as shown below (Fig. 5).

**Plate 7:**
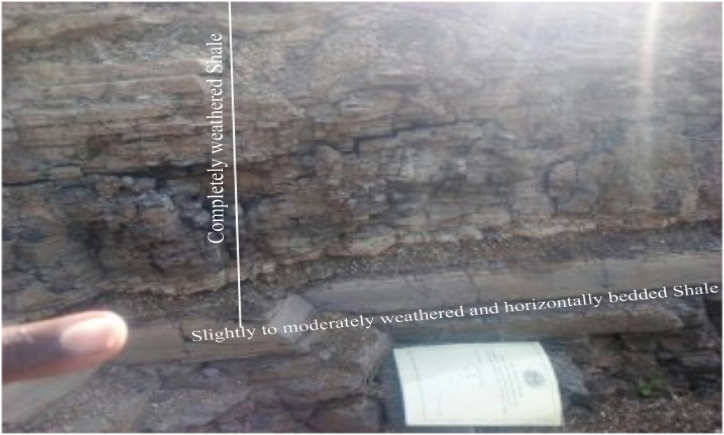
Stream cut exposures of horizontally bedded moderately to completely weathered Shale. The individual thin layers are easily split from its bed rock formation. GPS reading of long. 37^ο^ 39 ^׳^ 58^״^, lat 10^ο^ 13 ^׳^ 89^״^ and alt. 2119 *m*.

**Shale:** Moderately to completely weathered fine grained sedimentary rocks of Unmappable stream cut exposures of shale formation is observed on locally limited area. The rock unit is horizontally bedded (Plate 7). Due to the fissility (the property of shale to split along layers) and laminations of shale formation, the individual thin layers are not tightly bonded and can be easily split from its bedrock formation. This reveals the time sequence of geological depositional history in the past.

Shale is relatively low permeable and can be rich sources of petroleum and natural gas.

## 3 Geological structures

The varieties of magnificent land forms and geologic structures are the implication for the area was suffered by intensive destructive and constructive power of nature in the past. As a result plenty of primary and secondary geological structures had developed on the different rock units. Largely extended six principal normal faults are well developed on the basaltic rock units. However, there is no proper measurement for the dip of faults due to absence of definite fault plane; three of them have roughly NNW and three of them have NNE trending and they are the mirror reflection of each other (Fig. 6) with about (80–150 and 50) m relative vertical displacement and dipping about 35°, 75° and 80° respectively.

Joints exist in all rock masses in various degrees of vertical penetration and lateral extension. The systematic and nonsystematic fractures of rocks without appreciable displacement are more common in the study area. The systematic geologic structures mostly developed on the fresh Aphanitic Basalt and sandstone formation while nonsystematic ones are mostly observed on the weathered Aphanitic Basalt. Mostly systematic joints (columnar joints & bedding) are wide open space and closed aperture without infilling material, but the nonsystematic joints are mostly filled with soil materials.

These discontinuities reduce the quality of rock strength and facilitate slope instability. The dominant geological structural measurements recorded in the field using Brunton compass are illustrated in Table 1 below.

**Table 1:** The orientation of geological structural measurement data.

Lithologic unit	Geologic structure	Strike	Dip direction	Dip amount
Aphanitic basalt	Fault	006^ο^	096^ο^	33^ο^
		012^ο^	102^ο^	75^ο^
		021^ο^	111^ο^	80^ο^
		005^ο^	275^ο^	35^ο^
		012^ο^	282^ο^	75^ο^
		021^ο^	291^ο^	80^ο^
	Joint (columnar joint)	025^ο^		
		011^ο^		
		008^ο^		
		022^ο^		
Sandstone	Joint	042^ο^		
		010^ο^	–	
	Bedding	175^ο^	–	0^ο^
		005^ο^	–	0^ο^
Shale		085^ο^	–	0^ο^

**Rose diagram:** A circular frequency histogram which is used to plot the orientation of planar features (faults, dikes, joints, etc.). It is used for directional (azimuthal) data that shows the frequency distribution of strike azimuth of fractures. The most commonly observed joint and joint sets of trending, and three normal faults have been plotted (Fig. 6).

### 3.1 Geological correlation

Correlation is an important geological technique that provides information regarding the changes that have occurred in the history of the Earth. Generally, the relationships can be performed in one or two ways.

1. **Fossil correlation**: can be carried out by comparing the type of fossils found in various strata
2. **Physical correlation:** by comparing the physical characteristics of strata with each other and can be accomplished using a number of criteria the color, texture, and type of minerals contained within a stratum.

**Basics of geological correlation:** according to the Danish scientist [20]; the geological correlation recognized with three ideology of sedimentation. Those are **Superposition, Original horizontality** and **Lateral continuity.** Using those principles, the correlation of this study was done applying physical correlation (Fig. 7 [21]).

The potential exposure of sandstone unit is estimated from the respective altitudinal difference recorded from GPS reading in the field (Table S2 uploaded as a supplementary material). The regional and local 3D view of the study area is produced using surfer and global mapper software. All the geomorphologic set up of the study area is clearly illustrated on (Fig. 8a and b). This map used to extract information all about geological environment to select root road to be designed for accessing the geological construction materials.

## 4 Conclusion

Based on fieldwork mapping and descriptions, the geological map of the Chemoga sub river basin was prepared. Mostly ArcGIS software was used to generate various maps of the study area. Based on these approaches, the actual geological environment of the Chemoga sub river basin was illustrated on the map.

Based on field assessment, qualitatively and potentially desirable grade of sandstone and basaltic formations are well exposed. Specially left channel of Chemoga as well as left and right channel of Wuseta Rivers is illustrated by a good quality and potential exposures of sandstone formation. The massive sandstone formations are formed nearly vertical rock faces and pose extensive rock falls in unexpected condition and time which is difficult to conduct extraction and transportation processes. Therefore, to minimize hazards and fatalities, slope failure should be considered.

Due to weathering effect and impurities (manganese, iron oxidation) within the minerals of sandstone formation, it reveals in various colors, but the most dominantly observed fresh colors of sandstone formations are red and light yellow.

The horizontally bedded (stratified), thick, and genetically from similar deposits of sandstone formation of Chemoga river basin confirms (strengthen) that the sporadic (periodic) events of significantly high energetic transgression and regression processes had occurred in the past geological time. The sandstone formation is enclosed by six major consecutive normal faults which all of them reveal a strike of nearly NNE and NNW trending and they are the mirror reflection of each other.

Fresh to slightly weathered basaltic formation of rock units have well exposed. The rock unit is easily accessible and workable. On this field assessment, the columnar basalt is detected in topographically limit areas and forms nearly vertical faces moreover clay, conglomerate and Chert formations are identified (recognized).

Based on the geological field investigation, the following recommendations are made.

➢ Qualitatively and potentially, the sandstone formation exists in a desirable status, but not easily accessible (addressable) due to the natural barrier of cliff and deeply incised gorge formation.
➢ The local communities reflected their positive feeling for effective and durable road to be designed and put into action.
➢ The researcher has strongly recommended the investors to put in their capitals for the extraction potential occurrences of the geological construction materials, i.e. sandstone, the fresh and workable basaltic stones, aggregates and aesthetically attractive smooth and well graded gravels and boulders
➢ The area is continuously has being affected by surficial processes and highly vulnerable for gully erosion, flooding, sliding and rock falls. Therefore to design effective and durable roots of road, geological hazard assessment of the study area is significantly important issue.

## Funding statement

This research is funded by Debre Markos University.

## Data availability statement

Data associated with this study has been deposited at research gate and research square (https://doi.org/10.21203/rs.3.rs-718221/v1).

## Declaration of interest's statement

The authors declare that they have no competing interest and the research was funded by Debre Markos University.

## Author contribution statement

Mulusew Minuyelet Zewdie & Dawit Asmare: Conceived and designed the experiments; Performed the experiments; Analyzed and interpreted the data; Contributed reagents, materials, analysis tools or data; Wrote the paper.
